# Large-Scale Plantlet Conversion and Ex Vitro Transplantation Efficiency of Siberian Ginseng by Bioreactor Culture

**DOI:** 10.1155/2013/829067

**Published:** 2013-10-31

**Authors:** Jingli Yang, Shicheng Zhao, Changyeon Yu, Chenghao Li

**Affiliations:** ^1^State Key Laboratory of Tree Genetics and Breeding, Northeast Forestry University, Harbin 150040, China; ^2^Department of Crop Science, College of Agriculture & Life Sciences, Chungnam National University, Daejeon 305-764, Republic of Korea; ^3^Division of Bioresource Technology, College of Agriculture and Life Sciences, Kangwon National University, Chuncheon 200-701, Republic of Korea

## Abstract

To achieve large-scale low-cost ex vitro acclimatization of Siberian ginseng plants, heart- and torpedo-shaped secondary somatic embryos (SEs) induced from germinated SEs on agar medium were collected and then inoculated to 10-l bubble column bioreactor, respectively. For plantlet conversion, inoculation of torpedo-shaped secondary SEs was more effective than heart-shaped SEs. TS2 (culture of torpedo-shaped SEs in a bioreactor with a 2-week subculture interval) plantlets had a higher root number and leaf number and larger leaf area than did HS3 (culture of heart-shaped SEs in a bioreactor with a 3-week subculture interval) and HS2 (culture of heart-shaped SEs in a bioreactor with a 2-week subculture interval) plantlets. Of these converted plants, TS2 plantlets had higher survival rate (83.7%) and growth characteristics after transplantation in a simple shed covered with a 50% sunshade net only for 6 months. TS2 plantlets also showed significantly lower H_2_O_2_ content and significantly increased superoxide dismutase (*SOD*), glutathione peroxidase (*GPX*), and glutathione transferase (*GST*) expression levels as compared to HS2 plants when exposure to ex vitro conditions.

## 1. Introduction

Over the past decade, there has been a significant application of somatic embryogenesis in rapid multiplication of plants, which provided a possible from-laboratory-to-large-scale industrial production. However, the extensive use of somatic embryo (SE) is still restricted by its relative high production costs and low survival rate of in vitro plantlets during ex vitro transplanting [1]. During in vitro cultivation, plantlets grow in a unique aseptic microenvironment with special climatic conditions of high relative humidity and low light intensity to allow heterotrophic growth. These conditions may lead to changes in plant with abnormal morphology and physiology, which are often characterized by retardation in the deposition of epicuticular waxes and development of functional stomata associated with the low ability of the plantlets to regulate water loss, which can lead to losses during acclimatization [2]. Most of the management of environmental growth conditions has been focused on the ex vitro acclimatization stage; little attention has been placed in the management of environmental growth conditions of in vitro culture. Nevertheless, some studies demonstrated that partial desiccation treatment for early stage SEs could facilitate ex vitro establishment of SE-derived plantlets [1, 3].

Siberian ginseng (*Eleutherococcus senticosus*) is a medicinal woody plant species that is endangered because of overharvesting in its natural habitat. The cortical tissues of the roots, shoots, and leaves are used for various medicinal purposes and in subsidiary food production [4]. Conventional propagation by seeds and stem cuttings is very difficult because of the long-term stratification needed to induce germination of the zygotic embryos, as well as the low rooting frequency of cuttings [5]. Therefore, production of Siberian ginseng plantlets by somatic embryogenesis as an alternative method has been reported [6, 7].

The use of bioreactors is a technology suitable for large-scale plant production of Siberian ginseng [8]. We previously used 10-l bubble column bioreactors to culture Siberian ginseng secondary SEs, which resulted in a higher productivity than in suspension flask cultures [9]. Suspension-culture-propagated heart-shaped secondary SEs that were inoculated in bioreactors could germinate uniformly in plant growth regulators-free medium. Mass-produced germinated SEs attained 64.7% conversion frequency. About 90% of in vitro plants survived after acclimatization but only obtained under controlled temperature of 21°C, requireing very high production costs [9]. Thus, a key problem of production of high-quality plants to decrease the cost at ex vitro acclimatization stage needs to be resolved before widespread commercial application. Moreover, most work are restricted to increase the induction, germination rate, and conversion into plantlets, with little focus on the underlying physiological and molecular processes during acclimatization [6, 7, 10].

The aim of this study was to establish an efficient plant propagation and ex vitro acclimatization method for Siberian ginseng using bioreactor culture of agar-medium-developed secondary SEs and determine the effect of such conditions on the rate of plant conversion and subsequent ex vitro growth in a shed covered with a 50% sunshade net and the underlying physiological and molecular processes during acclimatization.

## 2. Materials and Methods

### 2.1. Induction and Development of Secondary Somatic Embryogenesis on Agar Medium

Germinated SEs of Siberian ginseng (*Eleutherococcus senticosus*) developed in bioreactor culture from highly cyclic somatic embryogenic-competent cell lines (see [9]) were used as initial materials for the induction of secondary SEs. Bioreactor culture-germinated SEs were transferred to 1/3 MS [11] agar medium containing 1% (w/v) sugar and 2.5% (w/v) gelrite (DUCHEFA, The Netherlands) without plant growth regulators (PGRs) and were cultured at 21°C, 25°C, or 29°C. Ten plantlets were cultured in each 500-mL plastic culture vessel containing 70 mL medium (adjusted to pH 5.8, autoclaved at 121°C for 15 min) and were cultured with a 16-h photoperiod at 36 *μ*mol m^−2^ s^−1^ (cool white fluorescent tubes). After 8 weeks, the induction frequency of secondary SEs cultured at the different temperatures was calculated.

### 2.2. Cultivation of Secondary SEs in Bioreactors

Clusters of heart- and torpedo-shaped secondary SEs were synchronously developed from germinated SEs cultured on 1/3 MS agar medium for 6–8 weeks as described (see [9]). These secondary SEs were initially separated by gentle chopping with tweezers and inoculated into 250-mL Erlenmeyer flasks containing 50 mL 1/3 MS liquid medium and 1% (w/v) sugar. About 3000 heart-shaped or 1000 torpedo-shaped embryos were cultured in each flask. Cultures were agitated at 100 rpm on a gyrating shaker at 21°C.

After adapting to suspension culture for 1 week, SEs were inoculated in 10-l bubble column bioreactors (30 cm × 15 cm) containing 8-L 1/3 MS liquid medium (see [9]). About 12000 heart-shaped or torpedo-shaped SEs were cultured in each bioreactor. The cultures were agitated at one volume of air per volume of medium per min at 21°C. The effect of medium subculture period (a 2- or 3-week interval between medium changes) on SE development was evaluated. After 4 weeks (for torpedo-shaped SEs), 6 weeks (for heart-shaped SEs with 2-week subculture intervals), or 9 weeks (for heart-shaped SEs with 3-week subculture intervals) of bioreactor culture, fresh weight, hypocotyls diameter and length, and primary root number of germinated SEs were determined. Each set of bioreactor treatment conditions was replicated in three bioreactors, and each experiment was carried out twice. Germinated SEs were placed into 500-mL plastic culture vessels containing 70-mL 1/3 MS agar medium (~200 SEs/vessel) and stored at 4°C before being transferred to conversion medium.

### 2.3. Comparison of Plantlet Conversion and Transplantation Ability

For plantlet conversion, germinated SEs were transferred to conversion medium, which was 1/3 MS agar medium containing 1% (w/v) sugar and 2.5% (w/v) gelrite. Ten germinated SEs were cultured in each vessel. To improve the conversion frequency, germinated SEs stored in the vessel were exposed to light conditions (16-h photoperiod at 36 *μ*mol m^−2^ s^−1^) for 10 days before transfer to conversion medium. After 6 weeks, plantlet conversion frequencies were determined.

Randomly selected in vitro plantlets were transferred to plastic boxes (35 × 55 × 15 cm) containing a mixture of autoclaved sand and potting soil (1 : 3, v/v). About 50 plantlets were cultured in a box. Boxes were covered with perforated polythene bags to maintain high humidity and removed after 4 weeks. Plants were grown in a shed covered with a 50% high-density polyethylene sunshade net. Each of about 1500, 1000, and 500 germinated TS2-, HS2- and HS3-derived plantlets were used for transplantation experiment, respectively. The survival rate, area of leaf, length, and fresh weight of whole plants and root systems were compared after transplantation for 6 months.

### 2.4. Comparisons of H_2_O_2_ Content of Transplanted Plants

Differences between TS2- and HS2-derived in vitro plantlets after 0, 1, 3, and 5 days soil transplantation were investigated at the physiological level. H_2_O_2_ content was measured by Zhang et al. [12] with some modification. Fresh leaves (0.5 g) were ground to powder in liquid nitrogen and extracted with 5 mL of 5% trichloroacetic acid (TCA) and 0.15 g activated charcoal. The mixture was centrifuged at 10,000 g for 20 min at 4°C. The supernatant was adjusted to pH 8.4 with 17 M ammonia solution and then filtered. The filtrate was used for determination of H_2_O_2_.

### 2.5. Quantitative Reverse Transcription PCR (RT-qPCR) Assay of Antioxidant Gene Expression

Differences between TS2 and HS2-derived in vitro plantlets after 0, 1, and 3 days soil transplantation were investigated at the molecular level. Total RNA was extracted from leaf tissues using the CTAB method [13]. First-strand cDNA synthesized with 0.5 *μ*g purified RNA was reverse-transcribed with a reverse transcriptase kit (MBI Fermentas), and PCR was performed in a volume of 20 *μ*L, containing 10 *μ*L of 2 × SYBR premix ExTaq (TaKaRa Biotech, Dalian, China), 0.5 *μ*M of forward and reverse primers, and 2 *μ*L cDNA template (equivalent to 0.05 *μ*g of total RNA). The primer sequences of Siberian ginseng 18S rRNA (GenBank: AB080245.1), **β**-tubulin (GenBank: KC542394), and the previously defined stress inducible ROS-scavenging related genes (unpublished) *SOD* (GenBank: JQ365625.1), *GPX* (GenBank: KC542392), and *GST* (GenBank: KC542393) were given in Table 1 and amplified under the following cycling conditions: 10 sec at 95°C followed by 40 cycles of 5 sec at 95°C, 30 sec at 60°C, and 1 s at 78°C for plate reading. RT-qPCR was repeated three times for each sample on a DNA Engine Opticon 2 (MJ Research) following the manufacturer's recommendations.

### 2.6. Statistical Analysis

The data were analyzed with ANOVA using SPSS 16.0 for Windows (SPSS Inc., Chicago, IL, USA). Means that differed significantly were compared using Duncan's multiple range test at the 5% probability level.

## 3. Results and Discussion

### 3.1. Induction and Development of Secondary SEs on Agar Medium

**Figure 1:**
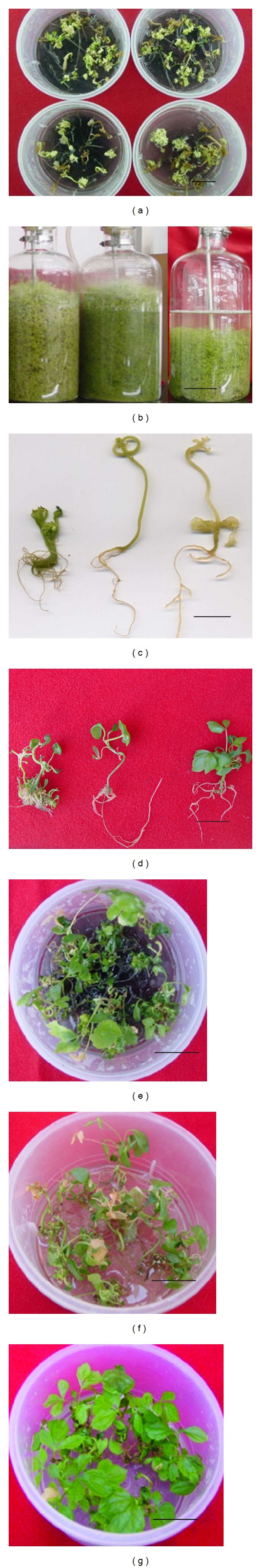
Bioreactor culture of agar medium induced heart-shaped and torpedo-shaped secondary somatic embryos of Siberian ginseng and plantlet conversion. (a) Numerous secondary SEs induced from germinated primary SEs on 1/3 MS medium without PGRs. Bar = 1 cm. (b) Germinated HS2 (left), HS3 (middle), and TS2 (right) SEs production in a 10 l bubble column bioreactor. Bar = 10 cm. (c) Germinated TS2 (left), HS2 (middle), and HS3 (right) SEs production in a 10 l bubble column bioreactor. Bar = 2 cm. (d) Plantlets derived from germinated HS3 (left), HS2 (middle), and TS2 (right) SEs. Bar = 4 cm. Plantlet conversion of germinated HS3 (e), HS2 (f), and TS2 (g) SEs. Bar = 2 cm.

After transformation of the germinated primary SEs from the bioreactors to solid medium, the frequency of secondary SE induction was significantly different among the three culture temperatures. At 21°C and 25°C, the induction frequency was 30% and 52%, respectively, whereas almost 90.6% of SEs induced secondary SEs at 29°C (Figure 1(a)), demonstrating that the higher temperature was better for secondary SE induction. Most secondary SEs gradually developed to the heart- and torpedo-shaped stage about 6 and 8 weeks after culture with no medium exchange, respectively. Further development of torpedo-shaped SEs was arrested, and relative water content decreased to about 54% after 8 weeks. The number of secondary SEs per explants varied significantly. About 20% of germinated SEs produced 100 to 1000 SEs per explants at 29°C.

### 3.2. Bioreactor Culture of Secondary SEs

To allow the SEs to adapt to the bioreactor culture environment, heart- and torpedo-shaped SEs were first inoculated into 250-mL Erlenmeyer flasks with liquid medium. After adapting to suspension culture in flasks for one week, secondary SEs were inoculated into bioreactors. When cultured in 1/3 MS medium, the SEs inoculated in bioreactors grew quickly, but the growth rate decreased after ~2 weeks of culture. The subculture period significantly affected SE growth. HS2 SEs grew normally and consistently with 2-week subculture intervals, but with a 3-week interval, the HS3 SEs grew slowly and then stopped until the medium was changed again. After subculture at 2-week intervals in bioreactors, heart-shaped SEs (referred to as HS2) germinated and needed 6 weeks for growth, whereas 9 weeks were needed with 3-week subculture intervals (referred to as HS3) (Figure 1(b)) (data not shown). Similarly, torpedo-shaped SEs germinated and grew fully after 4 weeks with a 2-week subculture interval (referred to as TS2; Figure 1(b)). The morphology of germinated SEs showed a significant difference depending on the different inoculation stages (Figure 1(c)). The hypocotyl length of germinated TS2 SEs was half of germinated HS2 and HS3 SEs, but fresh weight, hypocotyl diameter, and root number in the germinated TS2 SEs were all significantly higher than corresponding values for germinated HS2 and HS3 SEs (Table 2).

### 3.3. Plantlet Conversion

**Figure 2:**
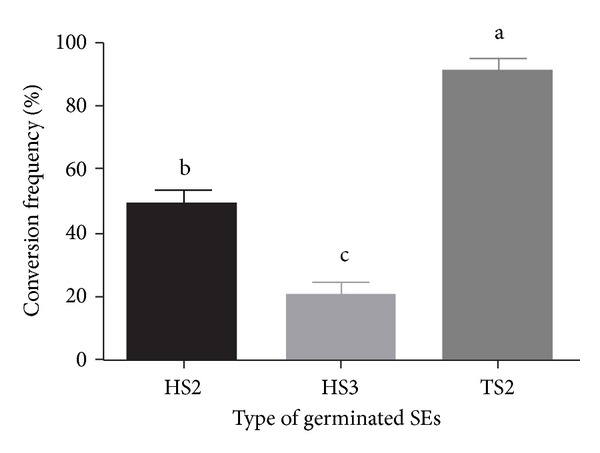
Effects of inoculation stage and medium exchange period on plantlet conversion of bioreactor cultured germinated SEs of Siberian ginseng after culturing on 1/3 MS medium for 2 months. Values with different letters in a column are significantly different according to Duncan's multiple range test at the 5% level.

Most bioreactor-cultured germinated SEs were pale yellow-green, possibly because of their high culture density, which would result in less absorbed light. Exposing the storage vessels with the SEs to light prior to culture on conversion medium resulted in quickly turning greened SEs produced. When exposing germinated SEs to light for 10 days, the conversion frequency extensively increased, and the conversion frequency of germinated TS2 SEs was significantly higher than that of HS3 and HS2 SEs (Figure 2). TS2 plantlets had a higher root number, leaf number, and larger leaf area than did HS3 and HS2 plantlets (Figures 1(d) to 1(g)) (data not shown). The leaf shape of TS2 plantlets was also more similar to that of seedling plantlets (Figures 1(d) to 1(g)). 

### 3.4. Comparison of Transplantation Ability of HS2, HS3, and TS2 Plants

**Figure 3:**
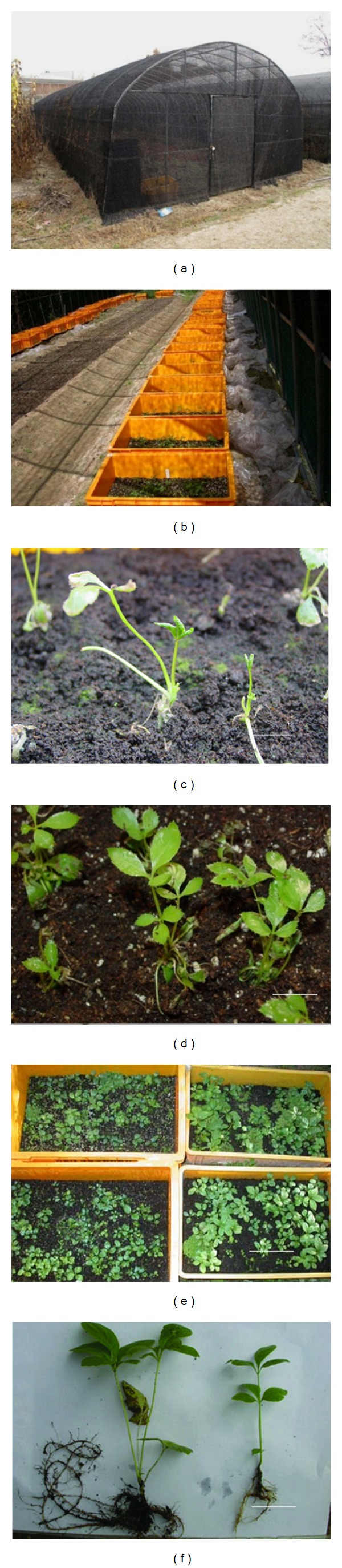
Transplantation of bioreactor cultured SE-derived plantlets of Siberian ginseng in simple sunshade shed. (a) Simple shed covered with a 50% sunshade net. Bar = 1.1 m. (b) In vitro plantlets were transferred to plastic boxes (35 × 55 × 15) containing a mixture of sand and soil. Bar = 20 cm. (c) HS2 plantlets 2 weeks after transplantation. Bar = 1 cm. (d) TS2 plantlets after transplantation for 2 weeks. Bar = 1 cm. (e) HS2 (left) and TS2 (right) plantlets after transplantation for 6 months. Bar = 16 cm. (f) TS2 (left) and HS2 (right) plantlets after transplantation for 6 months. Bar = 6 cm.

**Figure 4:**
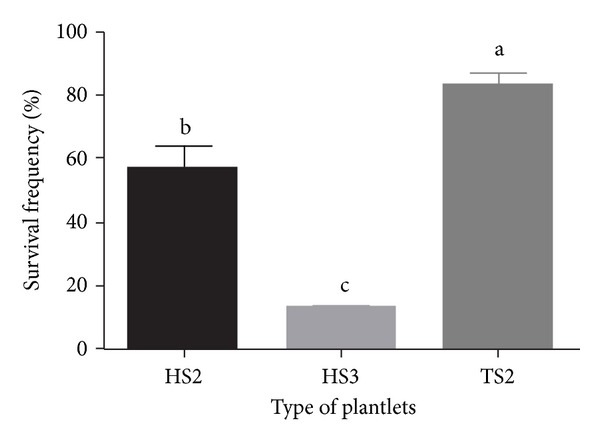
Survival frequency of HS2, HS3, and TS2 plantlet after ex vitro transplantation for 6 months. Values with different letters in a column are significantly different according to Duncan's multiple range test at the 5% level.

In vitro plantlets were transferred to soil and grown in a shed covered with a 50% sunshade net (Figures 3(a) and 3(b)). After transplantation, the leaves of HS2 and HS3 plants wilted or fell off within 2 weeks, followed by new shoots and leaves forming at the shoot apex within 4 weeks (Figure 3(c)). On the contrary, most leaves survived and maintained vigorous growth for TS2 plantlets (Figure 3(d)). Only for three days, TS2 plantlets had formed new roots, demonstrating that they can adapt quickly to the ex vitro environment. The results showed that survival rate of TS2 plantlets was significantly higher than that of HS2 and HS3 plantlets (Figure 4). TS2 plantlets also have higher growth characteristics than HS2 plantlets (Figures 3(e) and 3(f); Table 3). Although the survival rate in this study was similar to it in our previous report [9], the cost fee of this shading shed method was relative lower, which would be of benefit to the commercial application of plantlet production, because the transplantation was completely performed in a simple sunshade shed. The previous reported method need an accurate temperature (21°C) environment for ex vitro acclimatization; therefore, it requires an instrument high demand of.

To achieve large-scale vegetative propagation by somatic embryogenesis, the biggest obstacle is the mass production of SEs with high plantlet conversion ability [14]. Previous reports of somatic embryogenesis and plantlet production in Siberian ginseng showed that SEs germinate easily but have a relatively low conversion frequency and ex vitro acclimatization difficulty [6, 7, 10]. In the present study, the main cause for the high conversion and acclimatization ability of SEs was probably due to their long partial desiccation process at earlier stages of development over 6 to 8 weeks. A partial desiccation treatment is a common method for improving the plantlet quality of fully developed SEs, which can be otherwise difficult to germinate [15]. In coniferous species, SE desiccation treatment is the primary means for improving conversion rates [14]. Roberts et al. [15] improved the mature postembryonic development of SEs of *Picea glauca* ×* P. engelmannii* by gradually lowering the high relative humidity to achieve partial desiccation, which decreased the germination time, synchronized stem and root development, and resulted in >90% conversion frequency. In addition, partial desiccation treatment also successfully applied on large-scale SE production of coffee, rubber, and citrus trees by RITA bioreactor with a temporary immersion system [16–18].

### 3.5. Decreased H_2_O_2_ Content and Enhanced Expression of Antioxidant Genes in TS2 Plants

**Figure 5:**
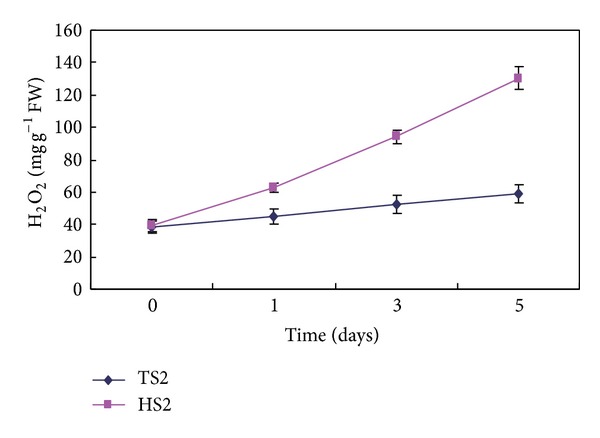
Comparison of H_2_O_2_ content between HS2 and TS2 plantlets after transplantation in a simple shed covered with a 50% sunshade net.

Abiotic stress may increase the level of reactive oxygen species (ROS) which is toxic to plant cells [18]. During desiccation, free radical scavengers may provide additional protection because the development of desiccation tolerance coincides with an increase in antioxidant gene expression in seeds [19, 20]. To investigate whether the decreased sensitivity to environmental stress in TS2 plants as compared to HS2 plants may be due to decreased ROS accumulation, the H_2_O_2_ content of TS2 and HS2 plants leaves was compared at initiation period of ex vitro transplantation. Under these conditions, H_2_O_2_ levels in TS2 plants were less than half of those in HS2 plants (Figure 5). Thus, TS2 plants may have more normal functional leaves and suffer less environmental stress as compared to HS2 plants. Our result strongly suggests that there is a correlation between the leaf damage and the imbalance of ROS production in early stage of transplantation.

**Figure 6:**
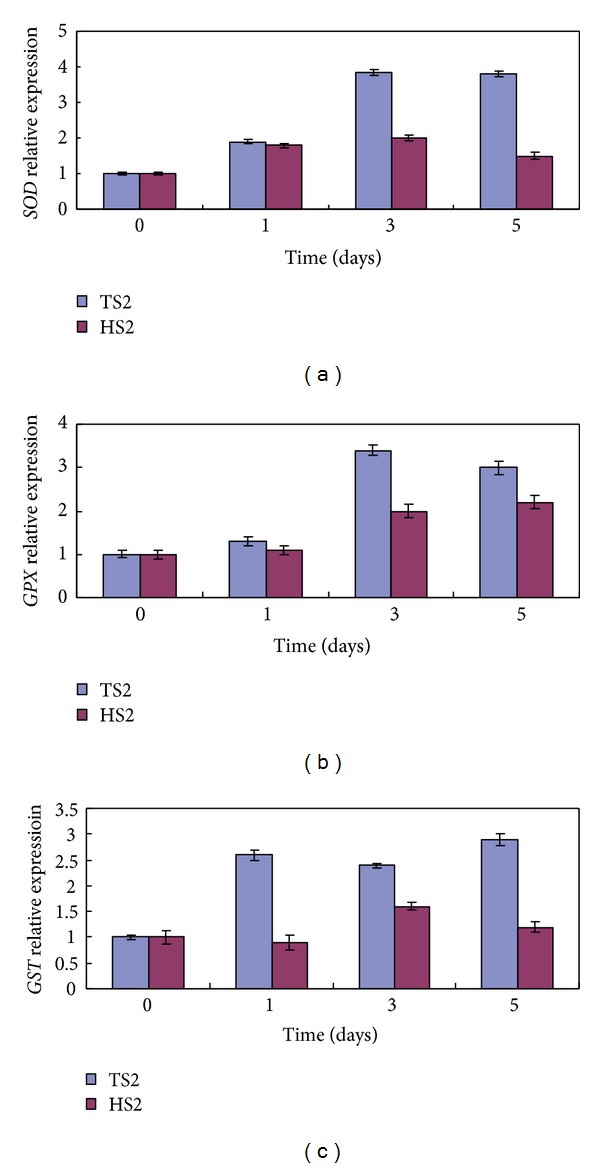
Comparisons of (a) *SOD*, (b) *GPX,* and (c)* GST *expression levels in HS2 and TS2 plantlet after transplantation in a simple shed covered with a 50% sunshade net by RT-qPCR.

It has been accepted that antioxidant defense systems—including nonenzymatic antioxidants such as ascorbate and reduced glutathione and enzymatic antioxidants such as *SOD* and *GPX*—play a crucial role in plant defense mechanisms against various stresses [21]. The expression levels of three stress inducible antioxidant enzyme genes *SOD* (GenBank: JQ365625.1), *GPX* (GenBank: KC542392), and *GST* (GenBank: KC542393) under ex vitro environment were compared between TS2 and HS2 plants using RT-qPCR. The results showed *SOD*, *GPX, *and* GST* expression levels in TS2 plants were increased significantly as compared to that in HS2 plants when exposed to ex vitro conditions (Figure 6). We hypothesized that ex vitro transplanted plant cells can induce expression of various antioxidant enzymes under environmental stresses, and the increased expression of these antioxidant enzyme genes in TS2 plants may contribute to the scavenging of ROS. Antioxidant enzyme genes' expression levels of HS2 plants also increased when exposed to environmental conditions in vitro, may be because the far more produced ROS could not protected leaves from oxidative damage.

## 4. Conclusion

An efficient micropropagation protocol for Siberian ginseng has been established. Development of secondary SEs to torpedo-shaped stage on agar medium prior inoculated to bioreactor culture was found to be beneficial for plant conversion and thus low the cost acclimatization of Siberian ginseng. TS2 plants were found to have higher environmental stress adaption ability compared to the HS2 plants. The protocol in the present study can be successfully exploited to widespread commercial propagation of Siberian ginseng elite clones.

## Figures and Tables

**Table 1:** Primers used for quantification of antioxidant enzyme genes by real-time PCR.

Primers	Sequence (5′ → 3′)
18S	F: 5′-GGTCGTGCCTCCGGCGCTGTTAC-3′
	R: 5′-CCTCTGACTATGAAATACGAATG-3′
*β*-tubulin	F: 5′-GGGAATTCGACTTCCATTCAAG-3′
	R: 5′-CAGGCTTCAACTTCCTCTTCTTC-3′
*SOD*	F: 5′-GGCCCAACTACAGTTACTGGAAG-3′
	R: 5′-GTGGCAGTACCATGTTCACCAACTG-3′
*GPX*	F: 5′-GCCTCACCAATTCAAACTACACAG-3′
	R: 5′-GGAGCGGCATTGCAACCATTCAC-3′
*GST*	F: 5′-CTCAAGCTAGGTTCTGGGCTGAC-3′
	R: 5′-GACAAGGATTACATCAACATACC-3′

**Table 2:** Effect of inoculation stage and medium exchange period on growth of SEs in 10-l bioreactor.

Stage of inoculated SEs	Medium exchange period (weeks)	Bioreactor culture period (weeks)	Fresh weight of germinated SEs (mg/SEs)^z^	Hypocotyl		Primary root number
				Diameter (mm)	Length (cm)	
Heart-shape^x^	2	6	66^a^	1.2^a^	5.0^a^	4.3^a^
Heart-shape	3	9	225^b^	1.2^a^	4.2^b^	1.7^b^
Torpedo-shape	2	4	137^c^	2.5^b^	2.4^c^	9.1^c^

^x^About 12000 agar medium developed SEs were cultured in a bioreactor.

^z^Values with different letters in a column are significantly different according to Duncan's multiple range test at the 5% level.

**Table 3:** Comparison between plants obtained from HS2 and TS2 SEs after transplantation for 6 months in a simple shed covered with a 50% sunshade net.

Origin of plants	Leaf area (cm^2^)^z^	Plant height (cm)	Plant fresh weight (g)	Root system fresh weight (g)
HS2 SEs	5.5^a^	17.3^a^	7.3^a^	3.9^a^
TS2 SEs	1.7^b^	12.5^b^	2.8^b^	1.2^b^

^z^Values with different letters in a column are significantly different according to Duncan's multiple range test at the 5% level.
